# Facial subcutaneous tumor

**DOI:** 10.11604/pamj.2018.29.69.14166

**Published:** 2018-01-24

**Authors:** Aryé Weinberg, Andreas Eberhard Albers

**Affiliations:** 1 Prosper-Hospital, Department of Otorhinolaryngology, Head and Neck Surgery, Recklinghausen, Germany; 2 Charité Universitätsmedizin, Campus Benjamin Franklin, Department of Otorhinolaryngology, Head and Neck Surgery, Berlin, Germany

**Keywords:** Epidermal cyst, cutaneous metastasis, cutaneous B-cell lymphoma

## Image in medicine

A 66-year-old woman presented with a group of painless round-shaped solid lesions underneath her right eye measuring 3 x 1 cm (A). The lesions increased in size over the past 2 months. Apart form a curatively treated larynx carcinoma the patient was healthy. Histology form the excised (B) lesion revealed epidermal cysts. Epidermal cysts are benign subcutaneous tumors. They can appear in every part of the body. In the facial region, they are quite common. Nevertheless, as differential diagnosis cutaneous metastasis, merkel-cell carcinoma and cutaneous B-cell lymphoma should be considered among others. Therefore, complete excision and diagnosis based on histology is recommended.

**Figure 1:**
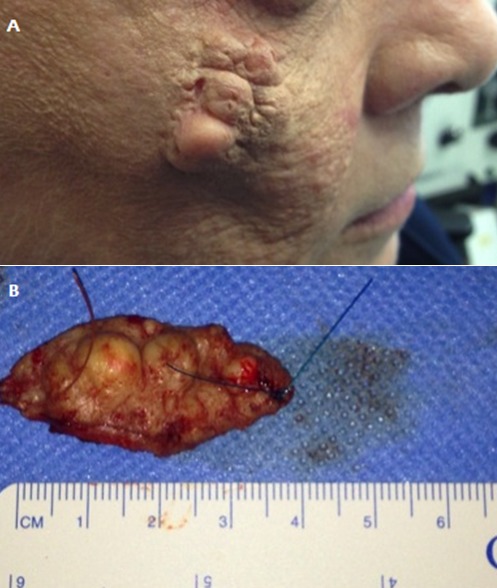
A) subcutaneous tumor; B) epidermoid cyst

